# Tracheal agenesis versus tracheal atresia: anatomical conditions, pathomechanisms and causes with a possible link to a novel *MAPK11* variant in one case

**DOI:** 10.1186/s13023-024-03106-z

**Published:** 2024-03-12

**Authors:** Mateja Pfeifer, Helga Rehder, Maria Gerykova Bujalkova, Christine Bartsch, Barbara Fritz, Cordula Knopp, Björn Beckers, Frank Dohle, Matthias Meyer-Wittkopf, Roland Axt-Fliedner, Alexander V. Beribisky, Manuel Hofer, Franco Laccone, Katharina Schoner

**Affiliations:** 1 Institute of Medical Genetics, Medical University of Vienna, Waehringer Strasse 10, 1090 Vienna, Austria; 2 Institute of Forensic Medicine, University of Zürich, Zurich, Switzerland; 3 Berlin School of Economics and Law (HWR), Berlin, Germany; 4 Institute of Human Genetics, Philipps-University of Marburg, Marburg, Germany; 5 Institute of Human Genetics, RWTH, Aachen, Germany; 6 Children’s Hospital St. Louise, Paderborn, Germany; 7 Department of Pediatrics, Children’s Center Bethel, University Bielefeld, Bielefeld, Germany; 8 Department of Gynecology and Obstetrics, University Clinic Oldenburg, Oldenburg, Germany; 9 Division of Prenatal Medicine and Fetal Therapy, University Hospital Giessen, Giessen, Germany; 10 Institute of Pathology, Fetal Pathology, Philipps-University of Marburg, Marburg, Germany

**Keywords:** Tracheal agenesis, Tracheal atresia, Congenital high airway obstruction sequence (CHAOS), VACTERL association, Sirenomelia, *MAPK11* variant, p38beta

## Abstract

**Background:**

In this study we aimed to describe the morphological and pathogenetic differences between tracheal agenesis and tracheal atresia, which are not clearly distinguished from each other in the literature, and to contribute thereby to the understanding and management of these conditions. Both tracheal agenesis and tracheal atresia represent rare disorders of still unknown aetiology that cannot be detected by prenatal ultrasound. If the affected foetuses survive until birth these conditions result in respiratory failure and in futile attempts to rescue the infant’s life.

**Results:**

Autopsies and genetic analyses, including singleton or trio exome sequencing, were performed on five neonates/foetuses with tracheal agenesis and three foetuses with tracheal atresia. Tracheal agenesis was characterized by absence of the sublaryngeal trachea and presence of a bronchooesophageal fistula and by pulmonary isomerism and occurred as an isolated malformation complex or as part of a VACTERL association. Special findings were an additional so-called ‘pig bronchus’ and a first case of tracheal agenesis with sirenomelia. Tracheal atresia presenting with partial obliteration of its lumen and persistence of a fibromuscular streak resulted in CHAOS. This condition was associated with normal lung lobulation and single, non-VACTERL type malformations. Trio ES revealed a novel variant of *MAPK11* in one tracheal agenesis case. Its involvement in tracheooesophageal malformation is herein discussed, but remains hypothetical.

**Conclusion:**

Tracheal agenesis and tracheal atresia represent different disease entities in terms of morphology, pathogenesis and accompanying anomalies due to a primary developmental and secondary disruptive possibly vascular disturbance, respectively.

## Background

The terms tracheal agenesis (TAG) and tracheal atresia (TAT) are not clearly distinguished from each other in the literature and are commonly referred to as TA, Tracheal agenesis is defined as absence of the sublaryngeal trachea [1–3] whereas tracheal atresia describes the congenital obliteration of the tracheal lumen leaving a fibromuscular streak without cartilages between the nonobliterated segments. TAG and TAT are rare life-threatening conditions with a prevalence rate of less than 1:50,000 and a male preponderance of m:f = 1:0,5 [4, 5]. TAG is most commonly associated with a narrow tracheo- or bronchooesophageal fistula (TOF/BOF), not avoiding polyhydramnios and fatal respiratory distress at birth but allowing drainage of lung fluid, thus preventing in utero development of pulmonary hyperplasia, consecutive diaphragmatic eversion and foetal hydrops, a condition referred to as “*c*ongenital *h*igh *a*irway *o*bstruction *s*equence”. CHAOS is not associated with a TOF or BOF [6, 7].

Concerning the position of the TOF/BOF, Floyd et al. [8] devised a classification, distinguishing between partial TAG with upper tracheal agenesis and with the lower trachea proximally communicating with the oesophagus (type 1), TAG with fusion of both main bronchi before communicating with the oesophagus (type 2) and TAG showing separate outlets of the main bronchi from the oesophagus (type 3). The frequency distribution recorded in 137 affected individuals was 33,6%, 49,6% and 16,8%, respectively [5]. The Faro classification on 40 tracheal agenesis cases from the literature includes 6 cases corresponding to TAT [9]. His types B, C and E correspond to Floyd’s type 3, 2 and 1.

In approximately 50% of cases, TAG is associated with prematurity and polyhydramnios [2]. As tracheal abnormalities cannot be visualised by ultrasound, it was recommended to perform a prenatal MRI in the presence of unexplained polyhydramnios and to consider tracheal or laryngeal atresia as possible cause of pulmonary hyperplasia [3, 10, 11]. The accompanying anomalies reported in 80–94% of the TAG cases mainly concern features of the VACTERL association [1–3, 5]. Evans et al. [1] assigned their pattern in 80 TAG cases to four subgroups according to the number of developmental fields involved. Radial limb defects were always right-sided. Lungs were always bilaterally bilobed or trilobed.

Perinatal intervention via "extrauterine intrapartum therapy" (EXIT) allows for targeted oesophageal intubation and life-saving postnatal oxygenation [5, 10]. Attempts at surgical neotrachealization of the oesophagus by vertical division or by internal or external stenting and oesophageal reconstruction have thus far been unable to provide long-term survival without neurological impairment [12, 13].

### Tracheal development

The etiology of TAG/TAT remains controversial. TAG has been induced by exposing rat embryos to adriamycin [14]. However, multiple genes have been described as being involved in tracheooesophageal development [15]. It was shown in mouse and Xenopus that preceding patterning of a dorsal and ventral foregut domain is a precondition for proper tracheooesophageal separation from the foregut, and that separation occurs by compartmentalization with locally increased proliferation of epithelial and mesenchymal cells along the lateral midlines [16, 17]. Patterning is established by expression of the transcription regulator Sox2 in the dorsal endoderm and of the thyroid transcription factor Nkx2-1 in the ventral endoderm [18–21]. Reduction of Sox2 leads to loss of dorsal patterning and—depending on the threshold levels—to oesophageal agenesis, and reduction of Nkx2-1 to loss of ventral patterning and thus to tracheal agenesis. In either condition the foregut remains a single tube with either oesophageal or tracheal wall structures and with connections to the stomach and lungs through a BOF or TOF. However, different signaling pathways and transcription factors establish and regulate *SOX2* and *NKX2-1* expression indicating involvement of numerous different genes in tracheooesophageal separation, e.g., *BMP4* and *SMAD1*, downregulating *SOX2* in the ventral foregut epithelium and Wnt2/2b inducing Nkx2-1+ respiratory progenitor ligands in the surrounding splanchnic mesoderm. In addition the ligands Shh, Ihh, and Wnt, whose expression is stimulated by retinoic acid (RA), are involved in the differentiation of splanchnic mesoderm into tracheal cartilage or oesophageal smooth muscle. It was also shown, that loss of RA and Hh, WNT2/2b- or BMP4- signalling results in loss of the tracheal phenotype [15–17, 22, 23]. However, no sequencing analyses have thus far been reported in affected humans.

We examined 5 cases of TAG and 3 cases of TAT in order to distinguish the anatomical structures and associations and discuss the possible causes and pathomechanisms of these conditions.

## Methods

Foetal autopsies, X-ray or MRI and thorough photographic documentation were performed on 3 neonates and 2 foetuses with TAG and 3 foetuses with TAT. Cases 1 and 3–8 had been sent for autopsy from different hospitals in Germany. Case 2 was derived from the Institute of Forensic Pathology in Zurich.

Cytogenetic analyses were performed according to standard procedures. Molecular analyses were performed on DNA derived from umbilical cord and foetal muscle (cases 1, 7–8), chorionic villi (cases 3 and 5), cultured amnion cells (case 4) and from parental blood (cases 1 and 5). The DNA isolated from different formalin fixed paraffin embedded (FFPE) probes of cases 2 and 6 was highly fragmented and not eligible for molecular testing. Array-CGH was performed using 4 × 44 K Human Genome CGH microarrays (Oxford Gene Technology, Begbroke, Oxfordshire, UK) and SNP arrays (GeneChip^®^, Affymetrix). Data were analysed using Agilent CytoGenomics software (Agilent Technologies, Inc., Santa Clara, CA). The probe sequence annotation was based on NCBI Build GRCh37 (hg19) of the human genome.

In cases 3, 4, 7 and 8 a singleton exome-sequencing (ES) was performed and in cases 1 and 5 a trio ES was performed. DNA samples were prepared following the workflow of the TruSeq Exome Library Kit (Illumina) or Twist Comprehensive Exome Panel (Twist Bioscience) for enrichment of exonic regions. The final library was paired-end sequenced on an Illumina NextSeq500 or NextSeq 2000 sequencer. Variants were evaluated using the program VarSeq Golden Helix® (Bozen, Montana) and were classified according to the American College of Medical Genetics and Genomics (ACMG) guidelines) [24]. A copy number variation (CNV) analysis of ES data by a module from VarSeq software was performed. The potential effects of the Met109Leu variant in MAPK11 were analysed using a structure of this protein (PDB ID: 3GP0) [25] already determined by X-ray crystallography in PyMol [26].

## Results

### Clinical data of TAG cases 1–5 (s. Table 1)

In none of the cases tracheal agenesis had been recognized prenatally. The pregnancies had been uneventful in the neonates of cases 1–3 except for late polyhydramnios and premature rupture of membranes and for prenatally suspected ‘oesophageal atresia' in case 3. Thus, the obstetricians were completely unaware of the condition. Any attempts to intubate the trachea or to find the trachea for tracheotomy failed. The neonates died 1–4 h postpartum due to insufficient oxygenation by high pressure ventilation through an endo-oesophageal tube. In the foetuses of cases 4 and 5, ultrasound diagnosis of the associated malformations led to termination of pregnancy.

**Table 1:** Clinical data, morphological, cytogenetic and molecular findings

Features	Case 1	Case 2	Case 3	Case 4	Case 5	Case 6	Case 7	Case 8
Gravida/para	II/0	IV/I	II/I	II/0	I/0	ND	III/II	I/0
Gest. weeks/outcome	3rd trimester (PROM)	liveborn (S)	3rd trimester (CS)	3rd trimester (TP)	2nd trimester (IUFD)	2nd trimester (TP)	2nd trimester (TP)	2nd trimester (TP)
Weight	2136 g	3000 g	1560 g	1020 g	21.8 g	350 g	362 g	630 g
Length (CHL)	49.3 cm	50 cm	41 cm	39 cm	11.3 cm	22.5 cm	24.3 cm	29.8 cm
Prenatal US	normal	normal	Possible oesophageal atresia, no TOF	VACTERL association	omphalocele sympodia	CCAM	CHAOS	CHAOS and defect of left hand
Polyhydramnios	+	−	+	−	−	Oligohydr.	−	−
Hydrops	−	−	−	−	−	+	+	+
Tracheal malformation	TAG + BOF	TAG + BOF	TAG + BOFs	TAG +BOF	TAG + BOF	TAT cranial segment	TAT caudal segment	TAT caudal segment
Pulmonary hyperplasia, lung weight in grams (times the norm for GA)	− 32.5 (0.96)	− n.d	− 27.0 (0.89)	− 21.1 (0.83)	− 0.72 (0.2)	+ 17.9 (2.5)	+ 41.6 (5.8)	+ 58.0 (5.5)
Lung lobes (right/left)	2/2	3/3	2/2	2/2	2/2	3/2	3/2	3/2
Disease entity/associated malformations	isolated TAG complex	TAG complex with right descend. aorta	VACTERL including AA, cardiac VSD, TAG, OF and right RLRD	VACTERL incl. SVD, ARA, card.-vasc. PLSVC, ARSA, TAG, OF, R(A)D, and right RLRD	VACTERL variant incl. SVD, ARA, cardiac AVSD, TAG, OF, RA, right RLRD, AG, omphalocele and sympodia	CHAOS and double left ureter, strangulating amniotic band	CHAOS and agenesis of corpus callosum	CHAOS and VSD, transverse defect of the left hand
BOF/TOF with Floyd classif.^a^	2	2	2+ “bronchus suis”	2	2	NA	NA	NA
BOF/TOF with Faro classif.^b^	C	C	C+ “bronchus suis”	C	C	G	G	G
VACTERL Evans subgroup^c^	NA	NA	3	4	4	NA	NA	NA
Family history/Consanguinity	−/−	−/−	−/−	−/−	−/−	−/−	−/−	−/−
Karyotype	46,XN	46,XN	46,XN	46,XN	46,XN	46,XN	46,XN	46,XN
CNV analysis (aCGH and/or ES)	− (aCGH and ES)	ND	− (aCGH and ES)	− (ES only)	− (aCGH and ES)	ND	− (aCGH and ES)	− (aCGH and ES)
Singleton- or Trio-ES/Result	Trio VUS in *MAPK11* (de novo)	ND	Singleton −	Singleton −	Trio −	ND	Singleton −	Singleton −

*aCGH* array comparative genomic hybridisation, *A(R)*A ano(rectal) atresia, *AG* absent external genitalia, *ARSA* aberrant right subclavian artery, *AVSD* atrioventricular septal defect (persistent AV canal), *(B)OF* (broncho)oesophageal fistula, *CCAM* congenital cystic adenomatoid malformation of lungs, *CHL* crown-heel length; *CS* caesarean section; *ES* exome sequencing; *GA* gestational age; *IUFD* intrauterine fetal demise; *NA* not applicable; *ND* not determined; *PLSVC* persistent left superior vena cava, *PROM* premature rupture of membranes, *RA* renal agenesis, *R(A)D* renal(a)dysplasia, *RLRD* radial (longitudinal) limb reduction defect, *S* spontaneous birth, *SVD* sacral vertebral defects, *TAG* tracheal agenesis, *TAT* tracheal atresia, *TLRD* transverse limb reduction defect, *TP* termination of pregnancy, *US* ultrasound, *VSD* ventricular septal defect, *VUS* variant of unknown significance

^a^Floyd et al. [8]

^b^Faro et al. [9]

^c^Evans et al. [1]

**Fig. 1:**
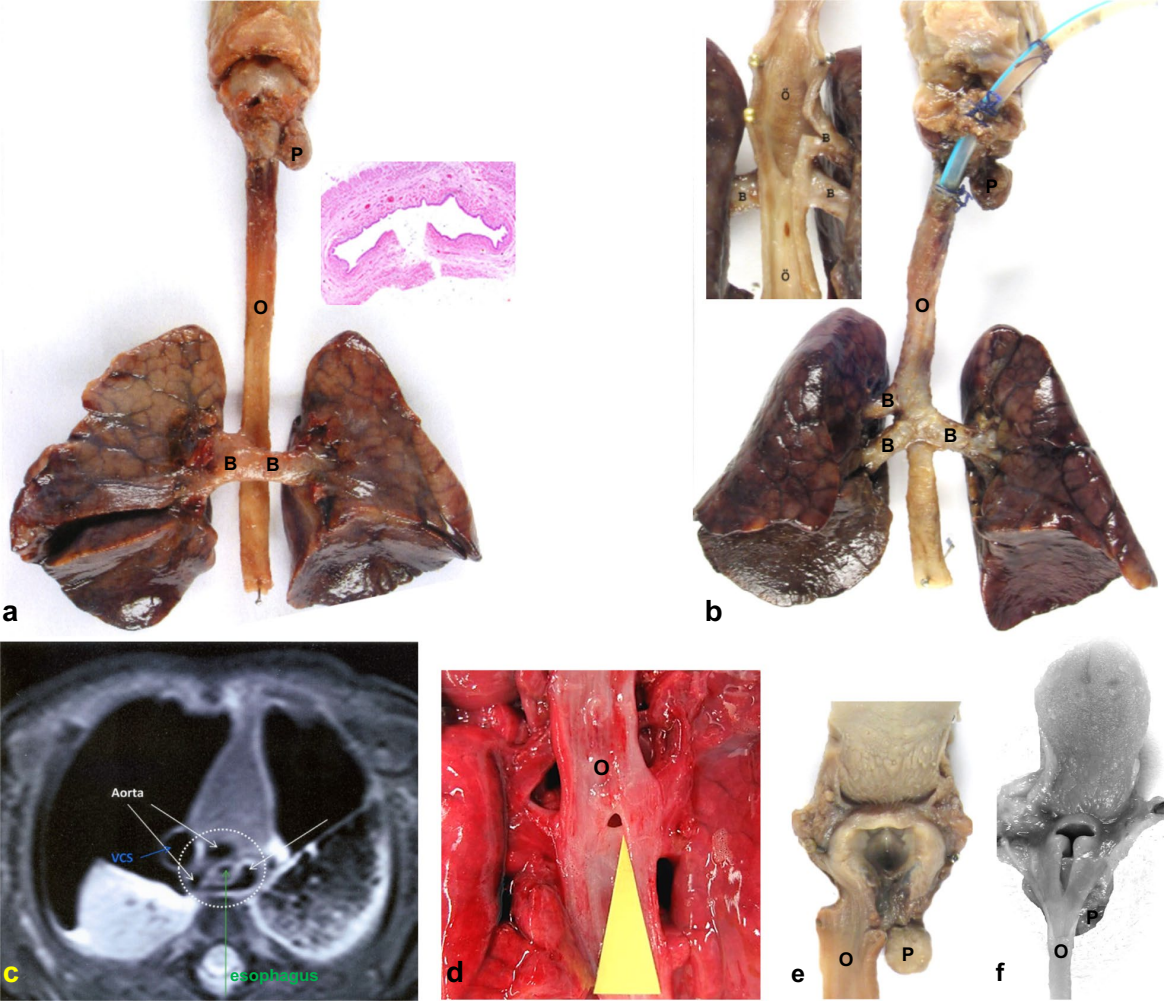
Tracheal agenesis—Case 1: Ventral view of the respiratory tract showing defect of the entire trachea except for a cartilage-free sublaryngeal pouch (P), with fused main bronchi (B), connected to the oesophagus (O) by a broncho-oesophageal fistula (BOF—TAG Floyd type 2) and with bilaterally bilobed lungs. Transversal section plane (H&E, 2,5) of the oesophagus with normally structured wall and without any tracheal components on histology (inlay) (**a**). Case 3: Ventral view of the respiratory tract with an absent trachea, sublaryngeal pouch (P), bilaterally bilobed lungs and a BOF connecting the fused right main bronchus (B) and left lower lobe bronchus with the oesophagus (O) and a second separate BOF connecting the left upper lobe brtonchus with the oesophnagus. Dorsal view of the opened oesophagus (O) and BOFs (inlay) (**b**). Case 2: Post-mortem MRI, axial T2 weighted image (T2WI) showing aorta (white arrows), vena cava superior (VCS) (blue arrow), oesophagus (green arrow) and lack of a trachea (**c**). Dorsal view of the internal surface of the oesophagus showing the opening to the BEF of only 1.5 mm in diameter (yellow arrow) (**d**). Case 4: View of the opened larynx from dorsal with the oesophagus (O) laid aside showing hypoplasia of the epiglottis and the tracheal pouch (P) dorsally opened (**e**). Case 5: Dorsal view of the larynx with a hypoplastic epiglottis, an intact position of the oesophageus (O) and a tracheal pouch (P) behind (**f**)

*Post-mortem examination of TAG cases 1–5* The foetuses/neonates showed an identical type of TAG with a sub-laryngeal blind pouch without cartilages, absence of the entire trachea and a tracheooesophageal fistula type 2 [8] (Fig. 1a, b, d–f). On histology the oesophagus showed a normal oesophageal wall without tracheal cartilages and with the mucous surface lined by a squamous epithelium (Fig. 1a inlay). In case 3, TAG was associated with an additional proximal BOF derived from the right upper lobe bronchus (Fig. 1b). A single tracheooesophageal tube was recognized by MRI in case 2 (Fig. 1c).

**Fig. 2:**
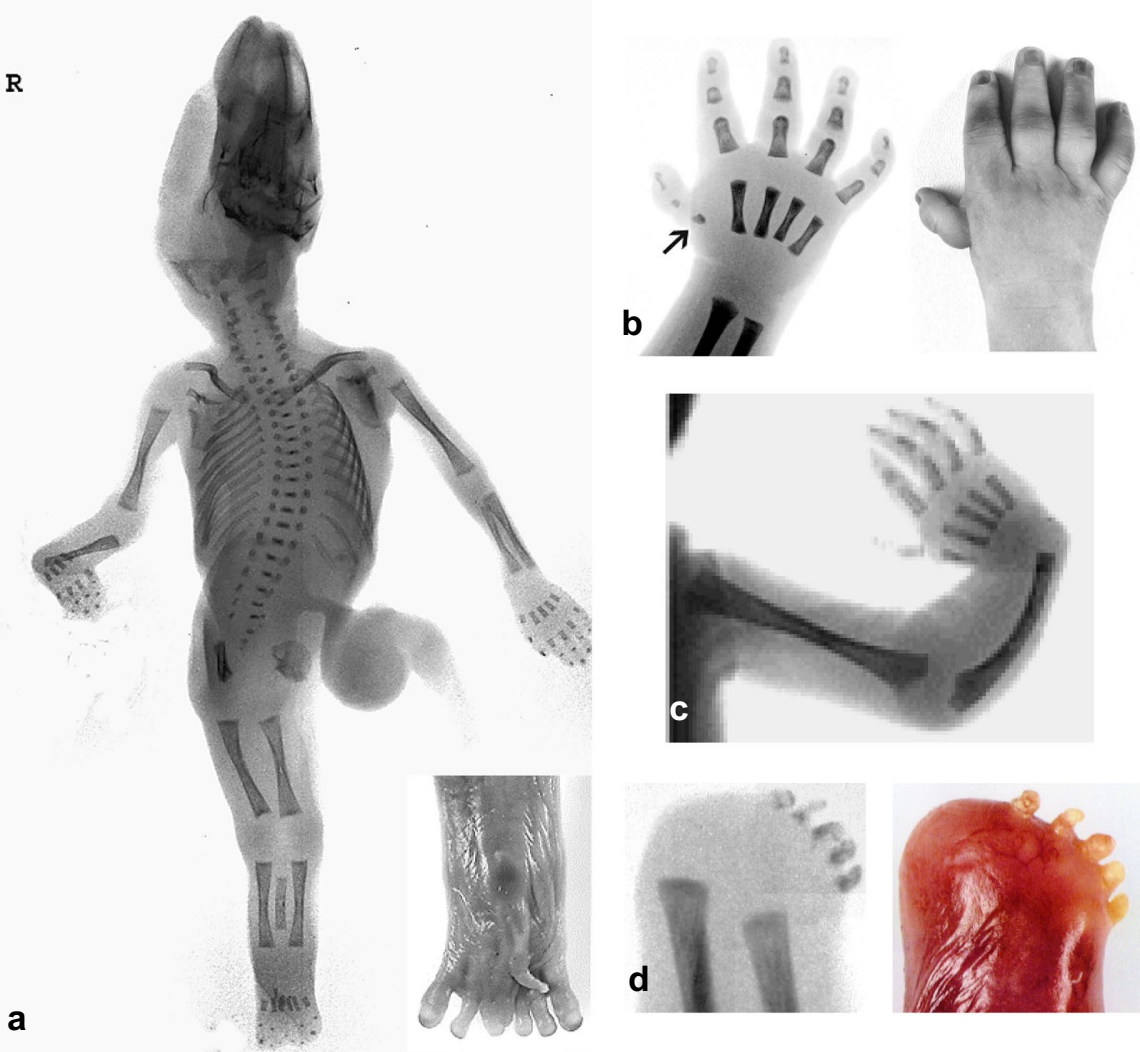
Limb reduction defects Case 5: AP-X-ray of the entire foetus showing sirenomelia with two femora and tibiae, a single fused fibula and foot, and eight metatarsals and toes (toes 4 and 5 fused) as well as radial deviation of the right hand due to radial aplasia combined with a defect of the 1st metacarpal and the thumb, furthermore scoliosis of the spine and only 11 ribs (**a**). Case 3: Right hand displaying a rudimentary 1st metacarpal and hypoplastic thumb appendage (**b**). Case 4: Radial adduction of the right hand due to absence of the radius and bowing of the ulna (**c**). Case 8: End of the slightly shortened left forearm displaying a distal transverse reduction defect including absence of the hand and hand bones with rudimentary five phalanges within five finger buds (**d**)

**Fig. 3:**
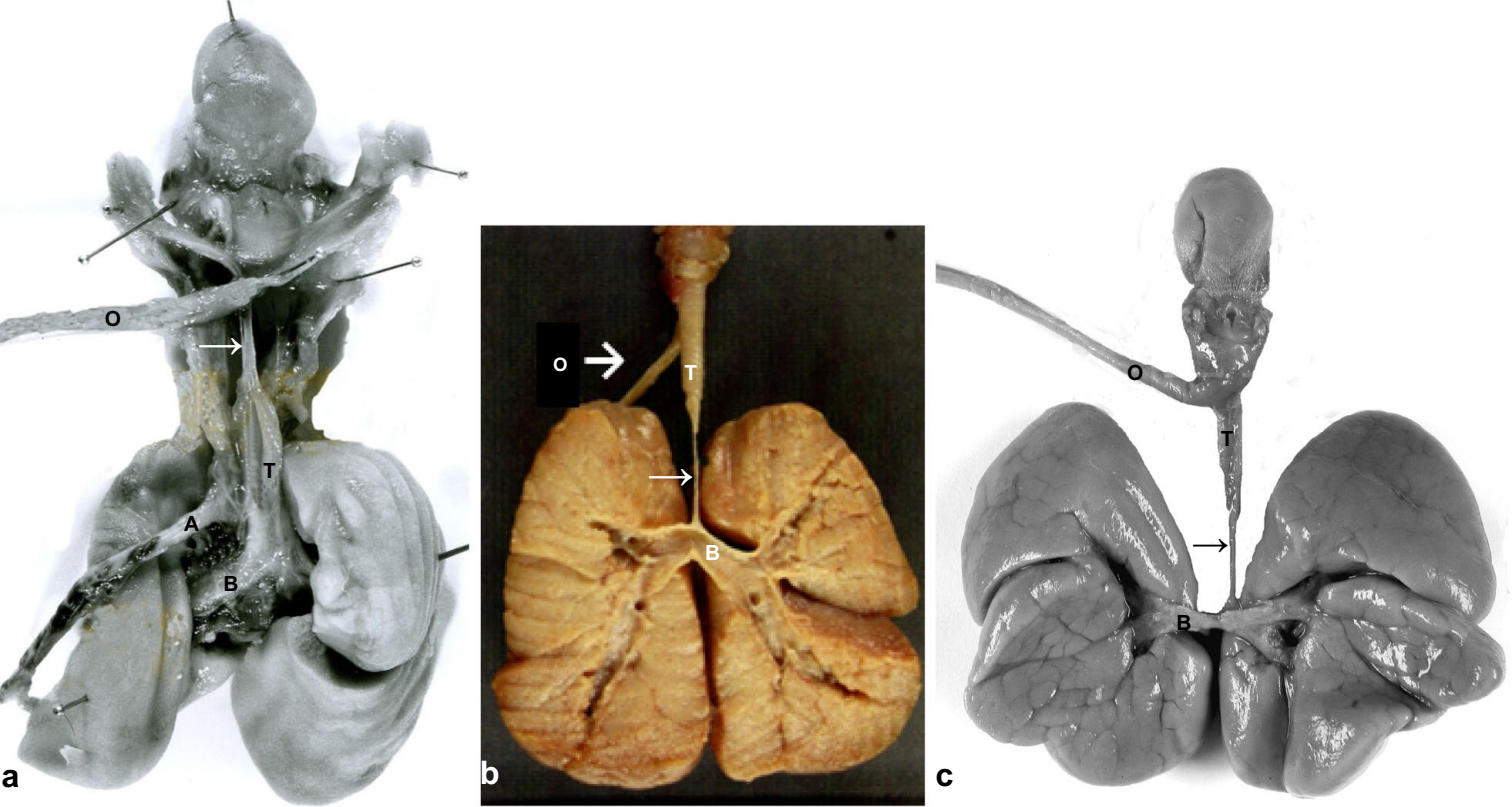
Tracheal atresia Case 6: Dorsal view of the respiratory tract with oesophagus (O) and aorta (A) laid aside, showing atresia with a streak-like remnant (arrow) of the upper trachea (T), dilated main bronchi (B) and hyperplasia of the lungs (**a**). Case 7: Ventral view of the respiratory tract with the oesophagus (O) laid aside displaying atresia with a streak-like remnant of the lower trachea (arrow), dilatation of the bronchi and hyperplastic lungs shown on the sagittal cut surface (**b**). Case 8: Dorsal view of the respiratory tract with the oesophagus (O) laid aside, atresia of the lower trachea (arrow), dilatation of the bronchi and hyperplasia of the lungs (**c**)

The associated anomalies diagnosed prenatally or at autopsy in cases 3 to 5 comprised vertebral, anal, cardiovascular, tracheooesophageal, renal and right -sided radial limb defects, (Fig. 3a–c), allowing diagnosis of a VACTERL association in cases 3–4 and, with respect to the sympodia in case 5, of a VACTERL variant (Fig. 2a-c). The TAG cases with VACTERL association as well as the isolated TAG in cases 1 and 2 were accompanied by either bilaterally two-lobed (cases 1 and 3–5) or three-lobed lungs with a right descending aorta in case 2. The sex ratio in cases 1–5 was m:f = 1:1.5.

### Clinical data of TAT cases 6–8 (s. Table 1)

CHAOS was diagnosed in cases 7 and 8 at 20 and 23 week’s gestation, while the hyperplastic lungs in case 6 had been interpreted as adenomatoid pulmonic hyperplasia at 19 weeks in 1994. Prenatal ultrasound revealed a transverse defect of the left hand in the foetus of case 8.

**Fig. 4:**
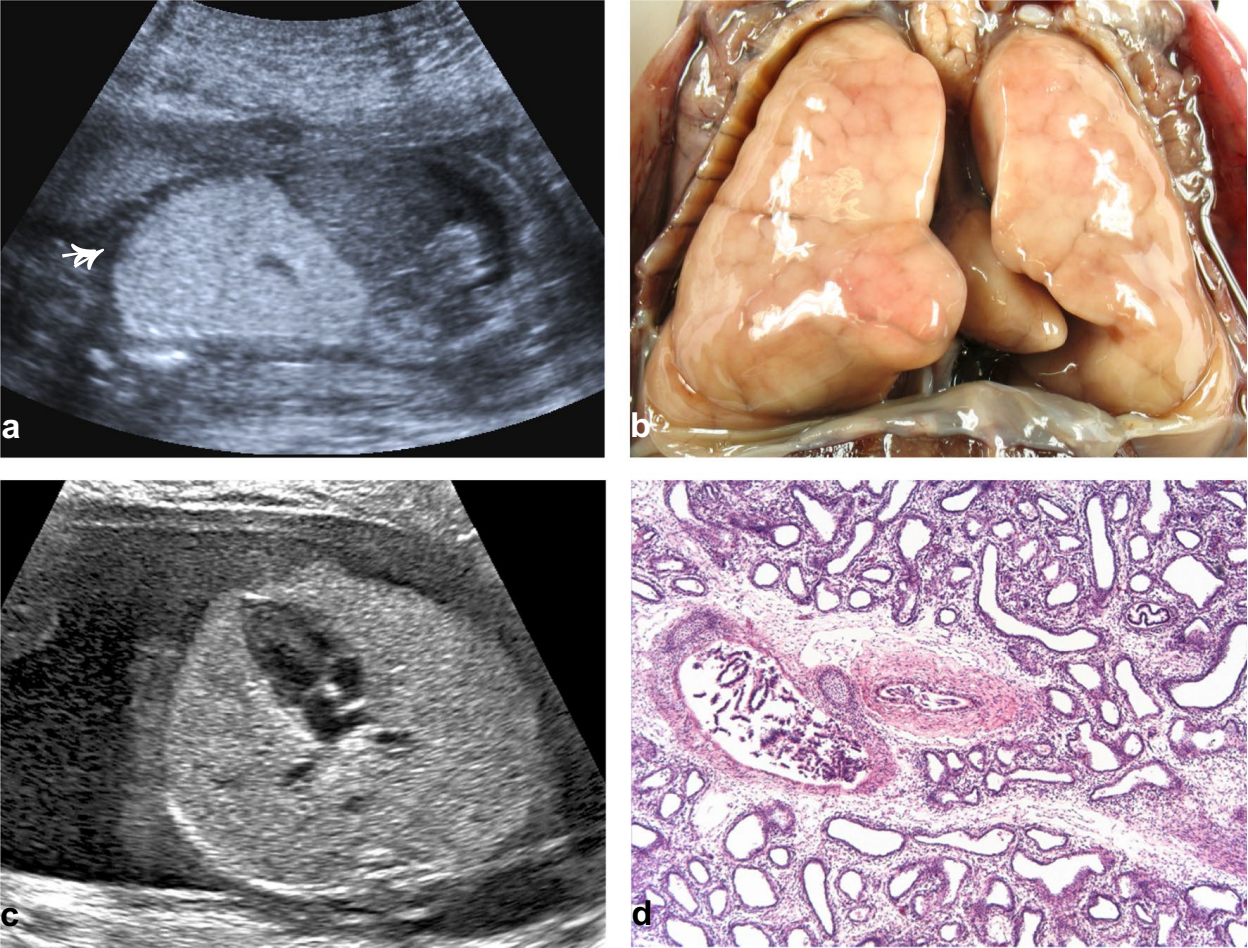
CHAOS due to tracheal atresia Case 7: Hyperplastic lungs on longitudinal section of the fetal thorax with inverted diaphragm in prenatal ultrasound (**a**), in situ (**b**), and on histology (H&E, × 5), showing normal lobulation and distended bronchi and alveoli (**d**). Case 8: Hyperplastic lungs on transverse section of the thorax in prenatal ultrasound with compression of the heart (**c**)

*Post-mortem examination of TAT cases 6–8* displayed a long distance tracheal atresia leaving a narrow fibromuscular streak without recognizable lumen or glandular or cartilaginous components. The atretic segment was located in the cervical trachea in case 6 and in the thoracic trachea in cases 7–8 (Fig. 3a–c). All three foetuses showed airway distension distal to the obstruction with lung hyperplasia, inverted diaphragm and hydrops thus fulfilling the criteria of 'CHAOS' (Fig. 4a–d). Associated anomalies comprised double left ureter and an amniotic strangulation in case 6, agenesis of the corpus callosum in case 7, and a VSD and transverse defect of the left hand with the absence of carpals and metacarpals and with rudimentary phalanges within five finger buds in case 8 (Fig. 2d). Sex ratio in cases 6–8 was m:f = 1:2.

In all 8 cases, parental consanguinity and a positive family history were excluded.

### Genetic analyses in cases 1–8 (Table 1)

**Fig. 5:**
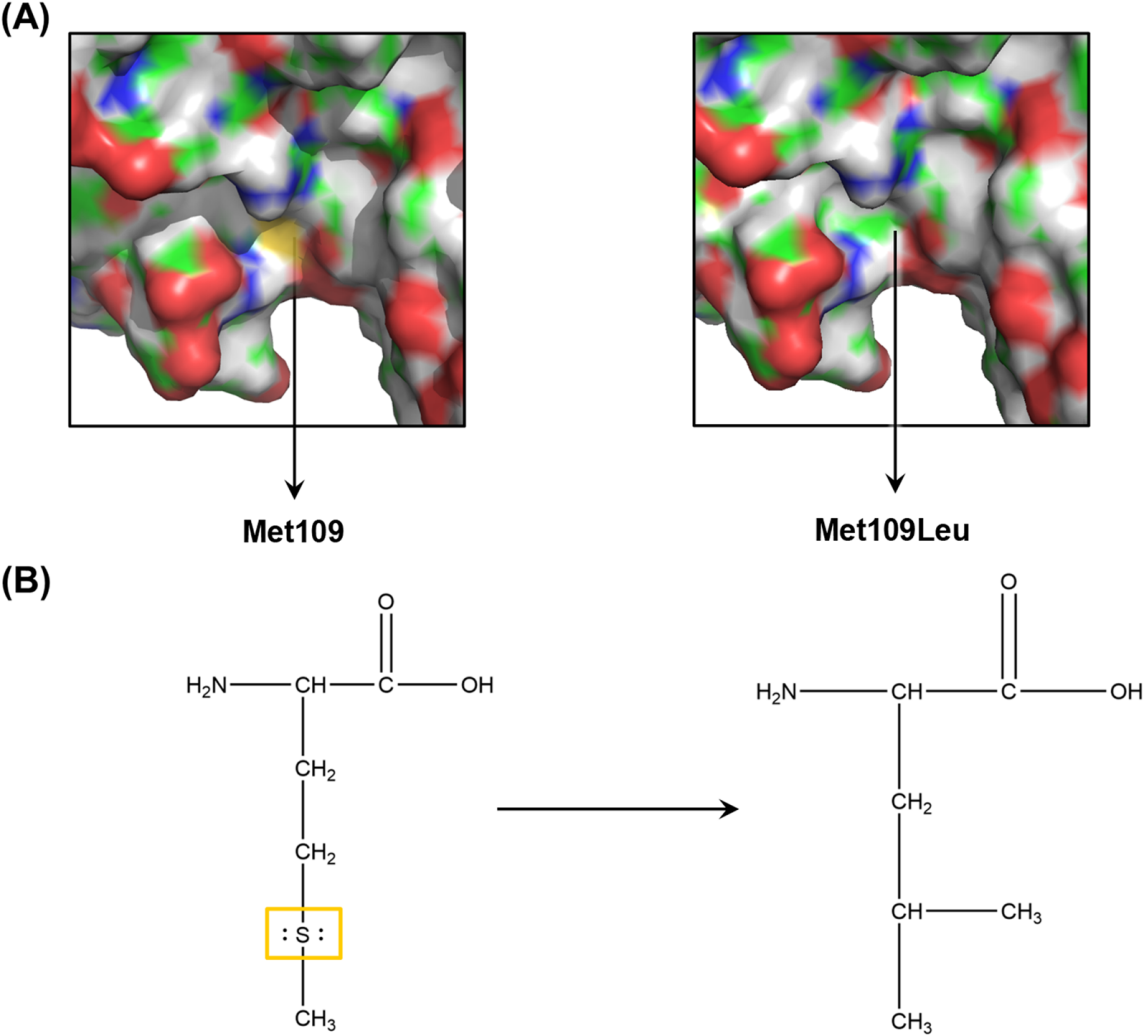
Structural analysis of the implications of Met109Leu substitution in MAPK11. The ATP binding site of MAPK11 with sulphur atom S_δ_ of Met109 marked in dark yellow (left) which is absent after a substitution Met109Leu (right). The crystalline structure of MAPK11 (PDB ID: 3GP0) was analyzed using the PyMOL software (**A**). Schematic representation of the structural differences between the methionine and leucine side chains. The sulphur atom S_δ_ of methionine along with its two electron ion pairs is highlighted in a dark yellow rectangle (**B**)

Karyotyping and CNV analyses showed normal results. Singleton ES in cases 3, 4, 7 and 8 and trio ES in case 5 revealed no disease-associated variants. Trio exome sequencing in case 1 revealed a heterozygous missense variant in the *MAPK11* gene of de novo origin (NM_002751.7:c.325A > T, NP_002742.3:p.Met109Leu; hg19). The identified variant site lies in a region which is referred to as a crossover connection in related structures [27], and next to the ATP-binding pocket (Fig. 5a) of the protein. The apparently conservative Met109Leu substitution [28] results in the loss of two ion electron pairs of methionine´s sulphur atom S_δ_ (Fig. 5b).

## Discussion

### Tracheal agenesis

In cases 1–5 TAG was characterized by a blind sublaryngeal pouch, most likely representing the former laryngotracheal diverticulum, by absence of a trachea, by a BOF of Floyd’s type 2 or Faro’s type C and by pulmonary isomerism. However, in case 3 there was an additional proximal BOF, connecting the right upper lung lobe bronchus with the oesophagus. A separate upper ‘tracheal bronchus, a so called ‘pig bronchus (bronchus suis)’, occurs with an incidence of 0.2% in humans [29]. Based on the ~ 200 TAG-cases described thus far, it was observed twice, including our case [30, 31].

Frequently associated malformations in TAG concern anomalies seen in the VACTERL association. According to Evans' subclassification, considering the number of developmental fields involved [1], our three TAG cases with VACTERL could be assigned to subgroups 3 (case 3) and 4 (cases 4 and 5), the latter representing a first observation of TAG associated with sirenomelia in terms of an extended developmental midline defect. We consider the ‘associated’ TAG to be part of the spectrum of possible VACTERL malformations, which is thus based on the same causes.

### Tracheal atresia

Contrary to TAG, TAT in CHAOS cases 6–8 presented with a trachea that is distally properly divided into two main bronchi, with accompanying non-VACTERL type malformations, with normal lung lobulation and with a sequence rather than an association. However, the trachea showed a long-distance obliteration of its lumen and lack of cartilages and mucous glands, leaving a residual fibromuscular cord between the non-obliterated segments. The obliteration affected either the cervical or the thoracic trachea. There was no TOF and thus no possibility for drainage of lung fluid, resulting in pulmonary hyperplasia and ultimately in CHAOS. Associated malformations included double ureters, absent corpus callosum, VSD and a terminal transverse LRD. These dissimilarities may indicate a differing etiology and pathogenesis of TAT compared with TAG. In this context the left-sided terminal transverse LRD in TAT as compared to the VACTERL type right-sided longitudinal LRDs in TAG is of interest. Preaxial longitudinal LRDs are often syndromic or part of an association, whereas terminal transverse LRDs are not, with a few exceptions such as Adams-Oliver syndrome, which shows additional vascularization and coagulation disorders resulting, e.g., in characteristic scalp defects. Transverse LRDs are generally sporadic and are thought to arise from teratogenic or vascular disruptions [32–34]. A vascular disruption could also be a conceivable cause of TAT, insofar as the tracheal blood supply involves different vessels. While the cervical trachea is supplied by the inferior thyroid arteries and its distal end by the bronchial arteries, the blood supply to the thoracic segment is variable and can involve the intercostal, subclavian, internal thoracic or innominate arteries [35]. The pattern of blood supply matches the pattern of TAT. However, the abovementioned vessels only supply the lateral and anterior tracheal walls. The dorsal wall receives its blood solely through oesophageal vessels [31]. This could explain the persistence of a fibromuscular cord in the atretic portion of the trachea.

### MAPK11 variant

In two cases each neither TAG nor TAT singleton ES revealed any pathogenic variants in OMIM-associated or relevant candidate genes. While a trio ES in the sirenomelic VACTERL foetus excluded de novo variants compared to the parental exomes, case 1 with the isolated TAG presented with a novel heterozygous missense variant of unknown significance (VUS) c.325A > T (p.Met109Leu) in *MAPK11*. This variant is neither listed in the current international databases, including gnomAD, nor has *MAPK11* been linked to any disease in OMIM. However, the residue in question, Met109, concerns a highly conserved region in MAPK11 protein homologues [27, 28]. Although it is only involved in a few intramolecular contacts (namely with Ala155), its location in a region that corresponds to the crossover region in related structures, and next to the ATP-binding pocket of the protein makes this residue crucial for the function of MAPK11. The seemingly conservative Met109Leu substitution [28], results in the loss of two ion electron pairs of methionine’s sulphur atom S_δ_ (Fig. 5b) which may affect proper ATP recognition by MAPK11 or distort its binding pocket, leading to compromised ATP hydrolysis and subsequently phosphorylation of downstream targets of MAPK11

*MAPK11* encodes the mitogen-activated protein kinase p38β. There are four p38 isoforms p38α-δ encoded by *MAPK14, MAPK11, MAPK12* and *MAPK13*. They are expressed ubiquitously in the mouse embryo with only p38α displaying additional extraembryonic expression in the placenta. p38 is involved in multiple signaling pathways determining the regulation of proliferation, differentiation and transcription and is activated through dual phosphorylation in response to post-inflammatory cytokines and environmental stress-induced signals [36, 37]. p38α and p38β have synergistic roles during mouse development and ~ 70% amino acid sequence identity, but p38α is expressed at higher levels in many cell types. This may explain the early embryonic lethality associated with intra- and extraembryonic *MAPK14* deletions and the postnatal nonviability owing to respiratory dysfunction associated with embryo-specific *MAPK14* deletions, as opposed to biallelic knockout deletions of *MAPK11* not resulting in aberrant phenotypes, presumably due to compensation of *loss of function* through p38 isoforms [37–39]

The regulatory function of p38 also applies to Sox2. Thus it was shown in melanoma cell lines that p38-dependent phosphorylation and thereby activation increases SOX2 stability and transcriptional activity [40]. Since Sox2 is expressed throughout the foregut epithelium and needs repression in the ventral foregut to establish Nkx2.1 expression [16], a causal relationship between a *gain of function MAPK11* variant promoting phosphorylation and thereby loss of tracheal patterning due to non-suppression of Sox2 seems theoretically conceivable. Unfortunately, we could not sequence the FFPE-DNA of case 2 in order to verify or exclude a *MAPK11* variant in our second child with *isolated* TAG. The lack of evidence of a pathogenic gene variant in our TAG cases 3 to 5 with accompanying VACTERL features may indicate that TAG is part of the VACTERL specific characteristics, a clinical picture affecting different developmental fields at different developmental periods for which no potential teratogenic or genetic alteration common to the majority of cases has yet been identified [41–43].

*In summary*, TAG and TAT represent different disease entities with respect to clinical features, aetiology and pathogenesis. TAG presents with absence of a trachea due to early lack of ventral tracheal patterning of and separation from the foregut, with a BOF/TOF preventing CHAOS, and with pulmonary isomerism; moreover TAG may present as an *isolated* malformation or as part of a VACTERL *association*. TAT is characterized by early degeneration of an existing trachea, by lack of a TOF causing CHAOS, by regular asymmetric lung lobulation, and by single non-VACTERL type accompanying malformations, and is possibly due to vascular disruption. An adverse effect of the identified *novel MAPK11* variant on tracheal development remains speculative. This could be explained by an increase in p38 activation and subsequent Sox2 stability in the ventral foregut. However, it should be kept in mind that the *MAPK11* variant if resulting in *loss o*f *function* may not be pathogenic due to compensation through p38 isoforms, and that p38β is not the predominant isoform, being expressed at lower levels in most tissues compared to p38α.

## Data Availability

Data that support the findings of this study are included in this article. Further enquiries can be directed to the corresponding author.

## Abbreviations

Array-CGH: Array-based comparative genomic hybridization
ATP: Adenosine triphosphate
Bmp4: Bone morphogenetic protein 4
BOF: Bronchooesophageal fistula
CHAOS: Congenital high airway obstruction sequence
CNV: Copy number variation
ES: (Whole) exome sequencing
FFPE probes: Formalin fixed paraffin embedded probes
gnomAD: Population database (genome aggregation database)
Leu: Leucine
LRD: Limb reduction defect
MRI: Magnetic resonance imaging
*MAPK11*: Mitogen-Activated Protein Kinase 11 gene
Met: Methionine
Nkx2-1: NKX2 homeobox 1 protein (= thyroid transcription factor1 TTF1)
p38beta: Mitogen activated protein kinase p38beta
PROM: Premature rupture of membranes
Shh: Sonic Hedgehog protein
SMAD1: Suppressor of mothers against decapentaplegic
SNP array: Single nucleotide polymorphism
Sox2: SRY-box 2 protein
TAG: Tracheal agenesis (agenesis = failure of development of an organ)
TAT: Tracheal atresia (atresia = closed or absent lumen of a hollow organ)
TOF: Tracheooesophageal fistula
VACTERL: Association of vertebral anomalies, anal atresia, cardiac septal defect, tracheooesophageal fistula, renal and radial limb defects
Wnt: Wingless/integrated protein
